# AFM-IR investigation of thin PECVD SiO*_x_* films on a polypropylene substrate in the surface-sensitive mode

**DOI:** 10.3762/bjnano.15.51

**Published:** 2024-05-24

**Authors:** Hendrik Müller, Hartmut Stadler, Teresa de los Arcos, Adrian Keller, Guido Grundmeier

**Affiliations:** 1 Technical and Macromolecular Chemistry, Paderborn University, Warburger Str. 100, 33098 Paderborn, Germany; 2 Bruker Nano Surfaces and Metrology Division, Östliche Rheinbrückenstr. 49, 76187 Karlsruhe, Germany

**Keywords:** AFM-IR, polypropylene, surface-sensitive mode, silicon oxide, thin films, XPS

## Abstract

Thin silicon oxide films deposited on a polypropylene substrate by plasma-enhanced chemical vapor deposition were investigated using atomic force microscopy-based infrared (AFM-IR) nanospectroscopy in contact and surface-sensitive mode. The focus of this work is the comparison of the different measurement methods (i.e., contact mode and surface-sensitive mode) with respect to the chemical surface sensitivity. The use of the surface-sensitive mode in AFM-IR shows an enormous improvement for the analysis of thin films on the IR-active substrate. As a result, in this mode, the signal of the substrate material could be significantly reduced. Even layers that are so thin that they could hardly be measured in the contact mode can be analyzed with the surface-sensitive mode.

## Introduction

Photothermal AFM-IR nanospectroscopy is a technique that combines the chemical information from infrared (IR) spectroscopy with the high spatial resolution of atomic force microscopy (AFM). For this, the sample is illuminated with a tunable IR laser [1]. When a suitable IR wavelength is chosen, resonant absorption of IR photons results in molecular vibrations in the material under investigation. This photon absorption also causes the thermal expansion of the material. The resulting photothermally generated tip–sample force is measured via changes in the deflection signal of the AFM cantilever. The correlation between the IR wavelength of the laser and the thermal expansion of the material enables the recording of IR absorption spectra with this technique which correspond to the spectra of bulk IR spectroscopy [2–4]. Compared to ATR-FTIR spectroscopy, AFM-IR provides a drastic improvement in terms of spatial resolution. In ATR-IR spectroscopy, the resolution is theoretically limited by λ/2, which corresponds to several µm [3]. In contrast, the development of new and powerful tunable IR laser sources, such as optical parametric oscillator (OPO) and quantum cascade lasers (QCL), enabled a nanoscale resolution of AFM-IR down to 10 nm [3]. Nowadays, the limit of the spatial resolution is given by the apex of the AFM tip.

One of the first AFM-IR demonstrations was reported in 2005 by Dazzi et al. [4], who presented AFM-IR spectra of single bacterial cells. Further on, this technique became more refined and found its way into many and highly diverse fields of application, including virology, DNA nanotechnology, polymer science, and materials science [5–9]. The importance of AFM-IR steadily grew over the past decade and its development has been described in detail in several review papers [1,3,9–10].

A general restriction of AFM-IR is its rather large depth of information, which depends on the sample structure and the chosen measurement mode such as contact mode or tapping mode. In general, the incident IR light excites a large volume of material beneath the AFM tip, and the tip–sample force generated by the thermal expansion of the total excited volume is detected. This makes it very challenging to characterize thin films with a small thermal expansion coefficient (e.g., inorganic oxides) deposited on bulk materials with a large thermal expansion coefficient (e.g., polymers). To tackle this problem, the surface-sensitive mode was developed.

### Surface-sensitive AFM-IR

**Figure 1:**
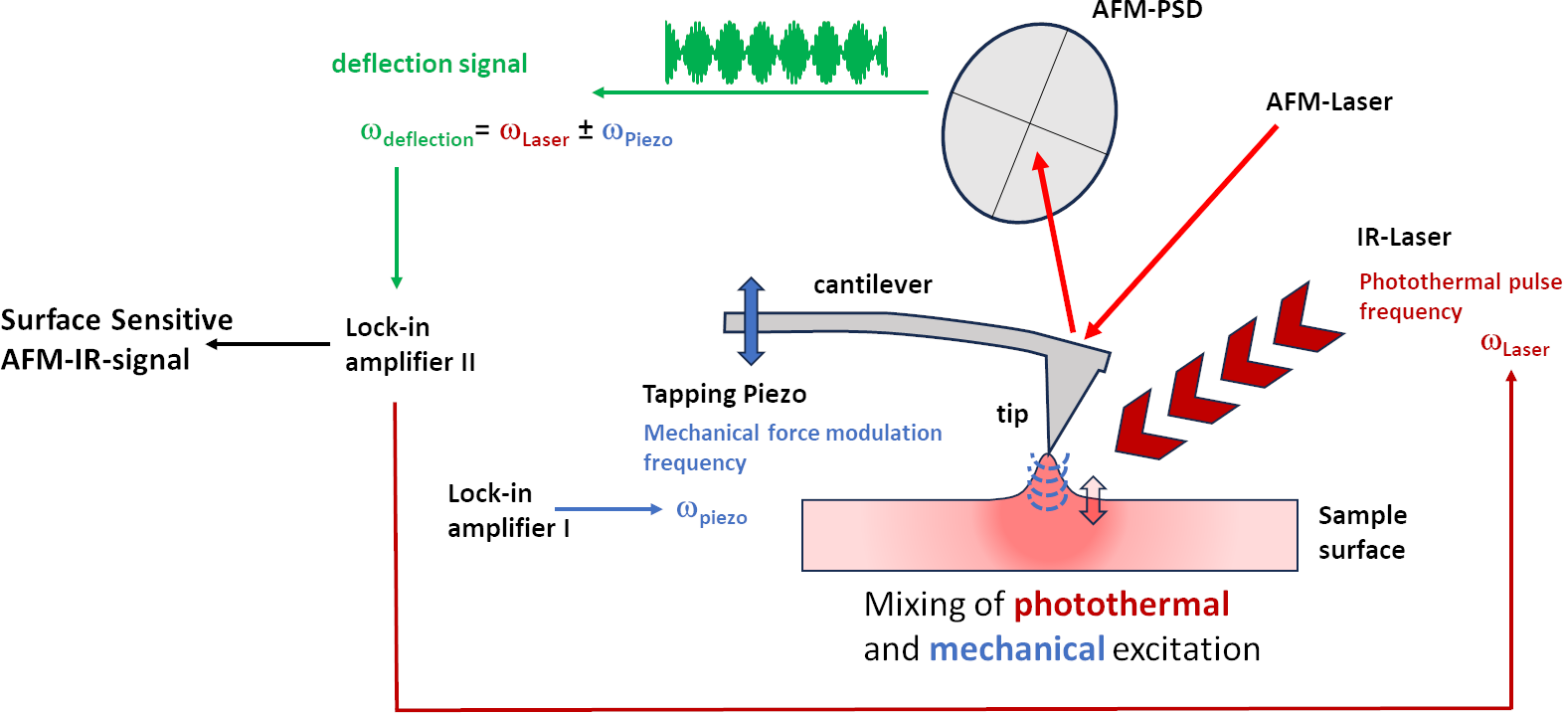
Detection scheme in surface-sensitive AFM-IR mode. Photothermal and mechanical surface excitation is achieved by driving the dither piezo and the IR laser source with two lock-in amplifiers. Nonlinear coupling of these processes in a small volume underneath the tip generates a surface expansion behavior containing the mixing frequencies, which are transferred into the cantilever deflection. Either the sum (C+M) or difference (C−M) frequency is chosen for detection. The chosen detection frequency should equal a mechanical contact resonance of the tip–sample contact for a sufficiently large signal level.

Surface-sensitive AFM-IR mode [10] operates in contact mode on the absorbing sample and utilizes nonlinear frequency mixing of IR-laser-induced photothermal and an additional dither piezo-induced mechanical surface excitation at two different frequencies schematically shown in Figure 1. Due to their different propagation characteristics in the material, the evanescent mechanical waves created by these two processes can only interact in a small volume directly beneath the tip (typically to a depth of less than 10–30 nm below the top surface).

As this is a higher-order nonlinear effect, the resulting tip–surface force is very small compared to that of classical contact-mode-based AFM-IR techniques, such as the ring-down method or resonance-enhanced AFM-IR, where the photothermal tip–sample force can be measured in a first-order detection scheme with or without additional resonance enhancement. However, it contains chemical information from a much larger depth (hundreds of nanometers to several micrometers). If the difference or sum of freely selectable laser pulse repetition frequency and mechanical modulation (i.e., drive frequency) in surface-sensitive AFM-IR mode equals a mechanical resonance of the tip–surface contact, sufficient IR signal enhancement can be obtained at this frequency (i.e., detection frequency). This is used to measure the IR signature of thin material sections close to the top surface without (or at least with severely limited) contributions from the bulk. In this description scheme, surface-sensitive AFM-IR shows similarities to the tapping-mode-based AFM-IR technique (i.e., the transduction of the IR absorption via nonlinear frequency mixing of photothermal and piezo-induced sample excitation). However, it is much more flexible in the choice of the drive and detection frequencies, as only the mixing frequency needs to match a system resonance and not the individual frequencies themselves.

Here, we use AFM-IR in the surface-sensitive mode to investigate thin silicon oxide films on polypropylene substrates. Polypropylene is widely used as packaging material [11] and in other industrial applications [12–14]; however, it is commonly known for its poor gas barrier properties [11,13]. Therefore, silicon oxide coatings are used to improve the gas barrier properties [13]. In this study thin coatings of SiO*_x_* were deposited by plasma-enhanced chemical vapor deposition (PECVD) in an oxygen-rich plasma process with hexamethyldisiloxane (HMDSO) used as monomer. With this process, the thickness of the coating can be controlled and homogeneous films can be produced [15].

## Experimental

### Materials and chemicals

The substrates used were polypropylene foils (LyondellBasell, Moplen Hp640J) onto which a thin layer of SiO*_x_* was deposited by PECVD. Therefore, the samples were placed onto the grounded electrode. The basis pressure was below 5 × 10^−5^ mbar and the working pressure ranged between 0.2 and 0.5 mbar. As the gas mixture, argon, oxygen, and HMDSO (98.5% purity, Sigma-Aldrich) were used in different ratios. First, the surface was pretreated for five seconds with an oxygen-rich plasma. For this step, the argon-to-oxygen ratio was set to 1:2. For the deposition of silicon oxide, the partial pressure of argon was set to 0.1 mbar and the partial pressure of oxygen was set to 0.3 mbar. The monomer partial pressure was set to 0.05 mbar. The high partial pressure of oxygen in a ratio of 3:1 promotes the formation of SiO*_x_* structures.

The film thickness was measured with a quartz crystal microbalance (QCM) during deposition. Samples were prepared with a SiO*_x_* thickness of 5 nm and 50 nm. The PECVD process is described elsewhere [16].

### Surface and thin film analysis

The infrared spectroscopic studies were performed using an Anasys NanoIR 3s system from Bruker Nano GmbH, Germany, equipped with a broadband Carmina OPO Laser (Angewandte Physik & Elektronik GmbH, Germany). Contact Mode NIR2 cantilevers from Anasys Instruments (PR-EX-nIR2-10) were used. The samples were first scanned in contact mode with a resolution between 512 × 256 and 256 × 256 pixels and an image size between 10 µm × 5 µm and 5 µm × 5 µm. In the AFM images, we selected a suitable spot to optimize the laser parameters. After the alignment of the laser, the pulse frequency was tuned. For the measurements in contact mode, the frequency was set to 69 kHz and, for the surface-sensitive mode, the second eigenmode of the cantilever was used as detection mode at 205 kHz. The drive mode was set to 845 kHz, which equals a higher contact resonance mode. The spectra were collected with a spectral resolution of 4 cm^−1^, and the phase-locked loop (PLL) was disabled for collecting the spectra.

In addition to these, measurements were performed comparing contact mode measurements, collected at a laser frequency tuned to the contact resonance mode of 771 kHz, and surface-sensitive measurements with the drive mode set to 764 kHz, the detection mode to 195 kHz, and the laser pulse was tuned to 566 kHz. Therefore, the sample with a 5 nm SiO*_x_* coating was used and, additionally to the AFM-IR spectra, a hyperspectral image was collected in contact mode.

In addition to photothermal AFM-IR measurements in contact mode and surface-sensitive mode, the surface was analyzed with near-ambient pressure X-ray photoelectron spectroscopy (NAP-XPS) using a NAP-XPS system with a Phoibos150 NAP analyzer from Specs Surface Nano Analysis GmbH. The setup has a µ-FOCUS 600 X-ray monochromator NAP source working with monochromatic Al Kα radiation at 1468.7 eV. The power was set to 50 W for all measurements. For the environmental charge compensation of the isolating polymer foils, the measurement was carried out in a 1.5 mbar N_2_ atmosphere. The survey spectra were recorded with a pass energy of 100 eV, while the core level spectra were taken with a 40 eV pass energy. The analysis was done using the software Unifit 2019 [17]. For all core level spectra, a Shirley type background was used. The Si 2p peak was fitted with doublet peaks with a Si 2p_3/2-1/2_ splitting of 0.6 eV [18].

## Results and Discussion

### XPS analysis

**Figure 2:**
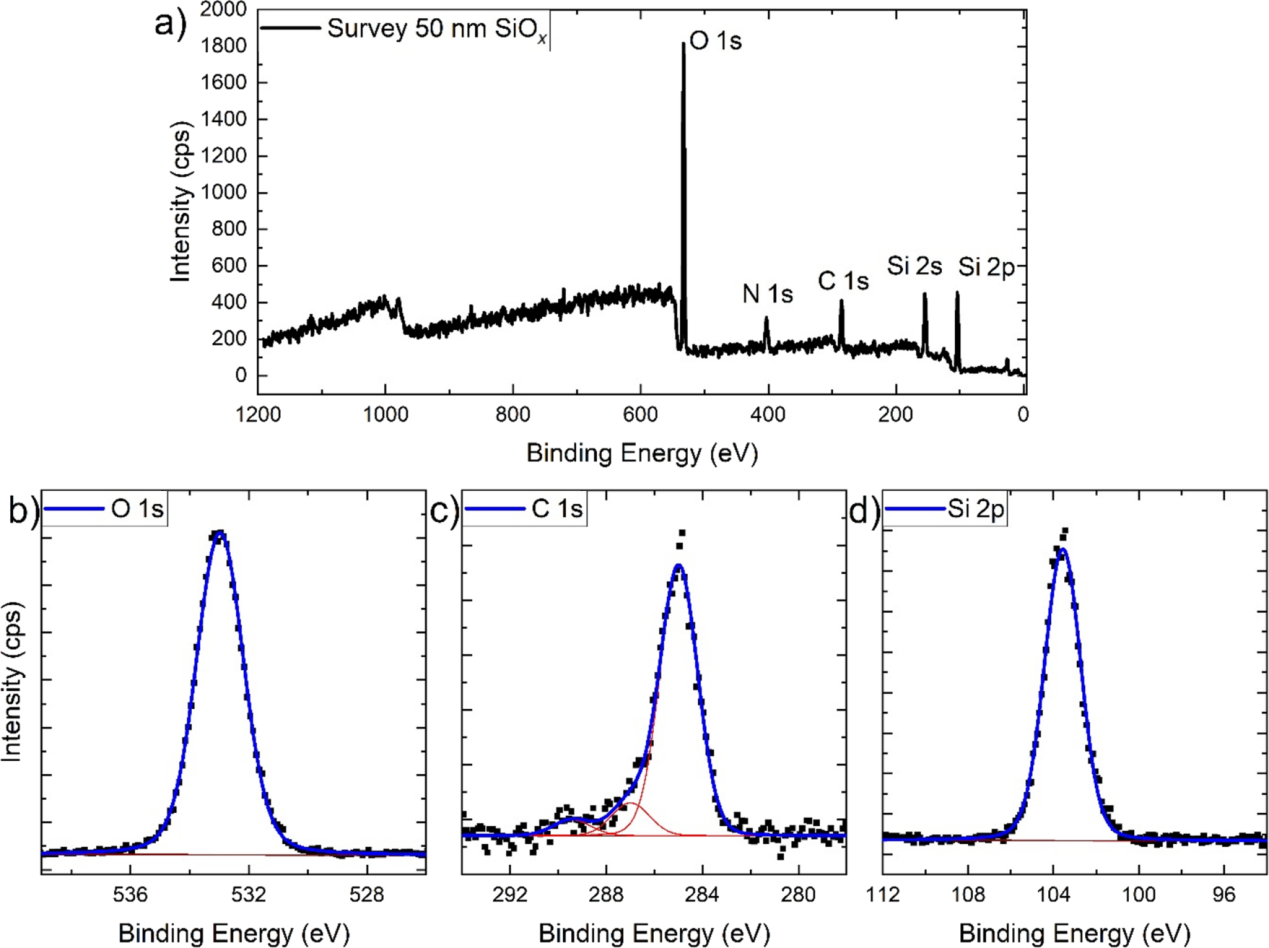
NAP-XPS survey (a) and high-resolution core level spectra of oxygen O 1s (b), carbon C 1s, (c) and silicon Si 2p (d) of the polypropylene foil covered with 50 nm SiO*_x_* measured in a 1.5 mbar N_2_ atmosphere for environmental charge compensation. Black dots represent measured data, while the blue lines are fits to the data that incorporate different components as indicated by the red lines.

**Figure 3:**
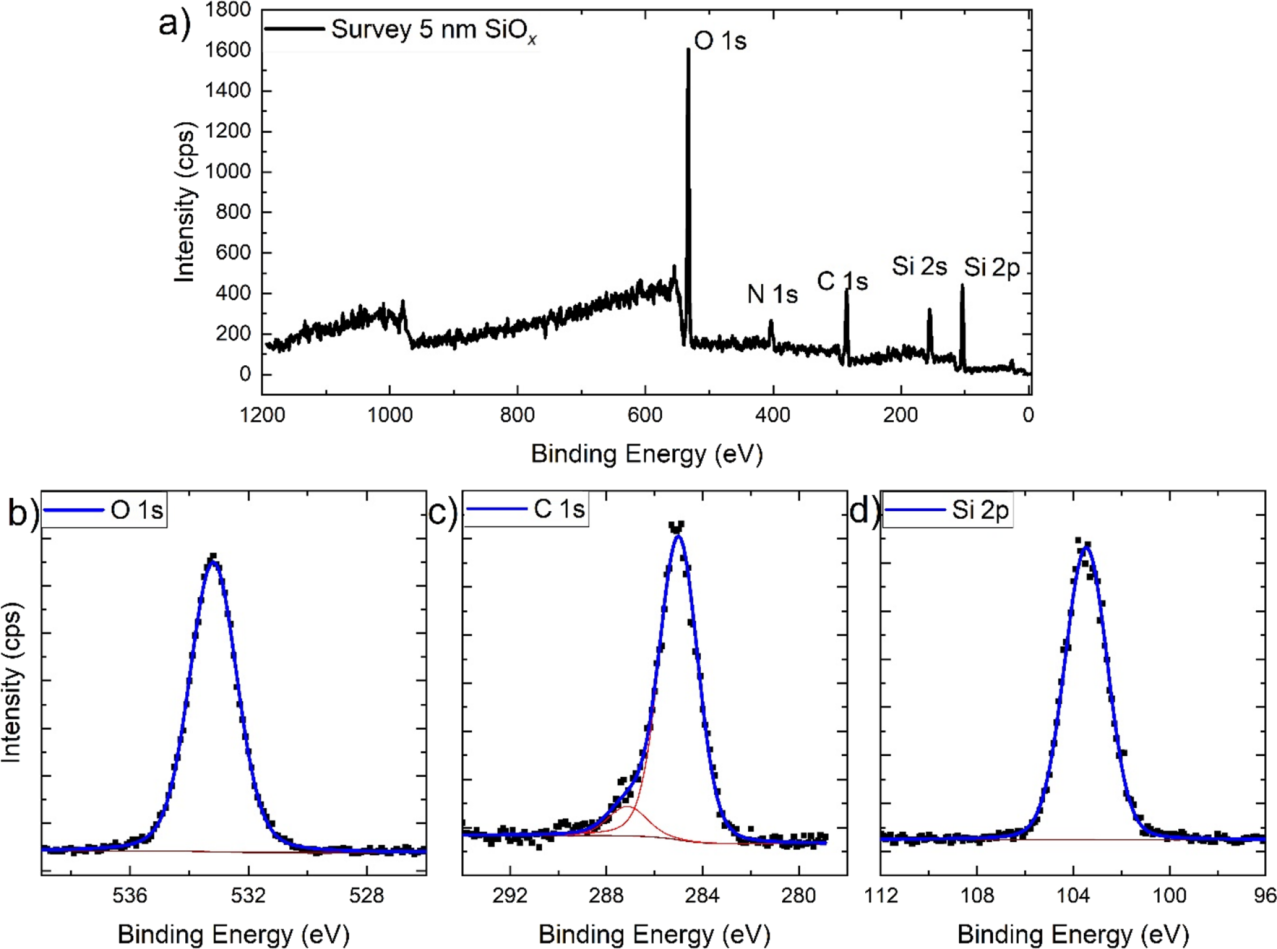
NAP-XPS survey (a) and high-resolution core level spectra of oxygen O 1s (b), carbon C 1s, (c) and silicon Si 2p (d) of the polypropylene foil covered with 5 nm SiO*_x_* measured in a 1.5 mbar N_2_ atmosphere for environmental charge compensation. Black dots represent measured data, while the blue lines are fits to the data that incorporate different components as indicated by the red lines.

The XPS data in Figure 2 and Figure 3 show that the deposition of silicon oxide thin films was successful. In the survey spectra, the peaks of oxygen, nitrogen, carbon, and silicon are visible. The N 1s peak originates from the nitrogen atmosphere which was used for the environmental charge compensation [18]. The C 1s peak can be assigned to adventitious carbon for the sample with a 50 nm SiO*_x_* layer. For the sample with a 5 nm SiO*_x_* layer, the C 1s is a mixture of adventitious carbon and the signal from the underlying polypropylene substrate.

The core levels of O 1s, C 1s, and Si 2p are shown in Figure 2 and Figure 3. In the figures, the spectra are arbitrarily charge corrected by fixing the binding energy of the Si 2p peak at 103.5 eV, which approximately corresponds to silicon oxide [19]. However, due to the impossibility to fix the BE scale in this case due to the nonconducting nature of the samples [20], the chemical identification of the oxide film is done by evaluation of the BE difference between the O 1s and Si 2p peaks. The O 1s–Si 2p distance is 429.6 eV for the 50 nm film and 429.9 eV for the 5 nm film. This is in good agreement with values found in the literature for the PECVD deposition of SiO*_x_* films from HMDSO/O_2_/Ar gas mixtures [21]. A comparison of the relative intensities of the O 1s and Si 2p peaks of the two samples (shown in Table 1) additionally supports the result that the chemistry of both SiO*_x_* layers is the same.

**Table 1:** Quantification of the surface composition of the silicon oxide films on the polypropylene foil.

Sample	O 1s–Si 2p (eV)	Relative areas [%]

50 nm SiO*_x_*	429.6	64
		36
5 nm SiO*_x_*	429.9	65
		35

### AFM-IR analysis

**Figure 4:**
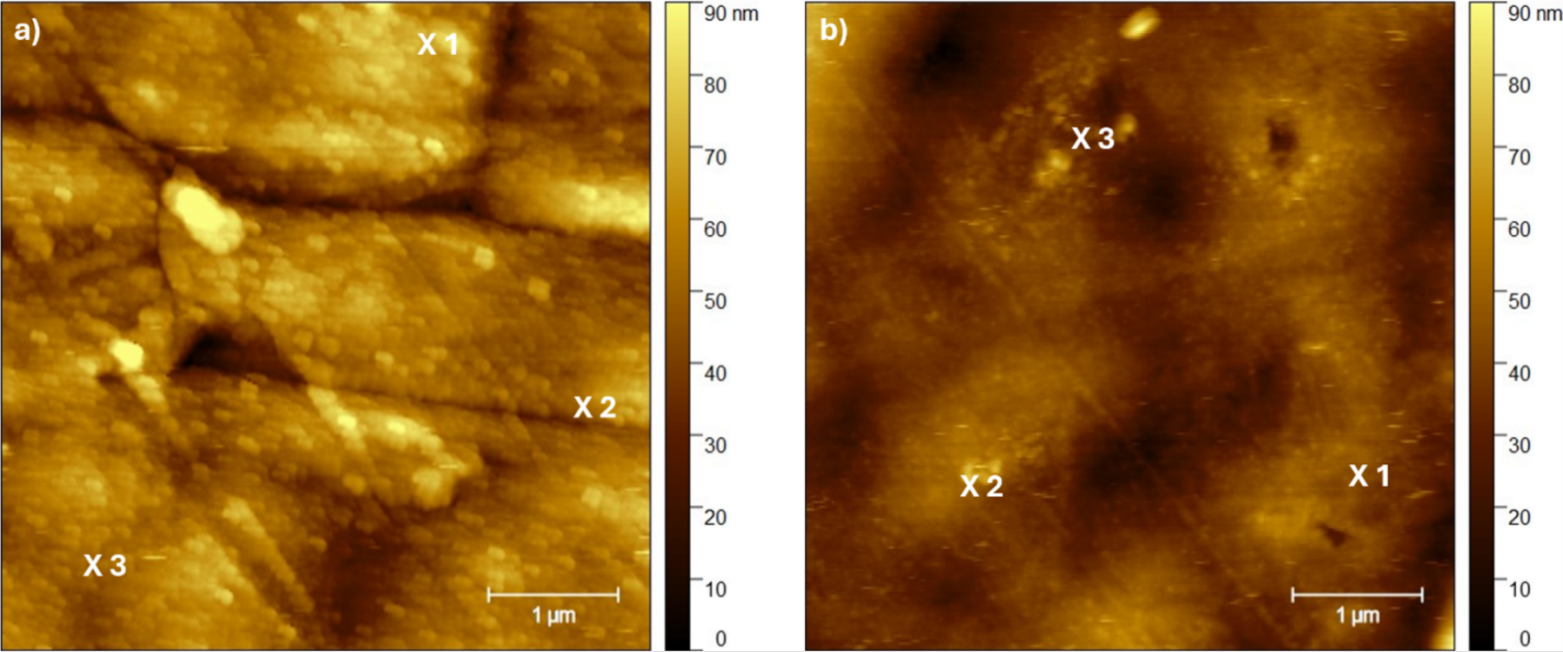
AFM height images of the polypropylene foil covered with 50 nm (a) and 5 nm SiO*_x_* film (b). The highlighted spots in the images represent the spots where the surface-sensitive AFM-IR spectra were recorded.

The prepared samples were analyzed by photothermal AFM-IR. First, AFM images were collected in contact mode to visualize the sample surface (Figure 4). In the image of the sample with the 50 nm SiO*_x_* layer (Figure 4a), a column-like grown SiO*_x_* film is visible. The surface became rougher. In addition to that, small cracks in the SiO*_x_* layer are recognizable. These cracks were induced by uniaxial stretching of about 10%. The topography of the 5 nm sample (Figure 4b) shows a smooth surface with elevations and valleys. These occur from the used polypropylene foil.

**Figure 5:**
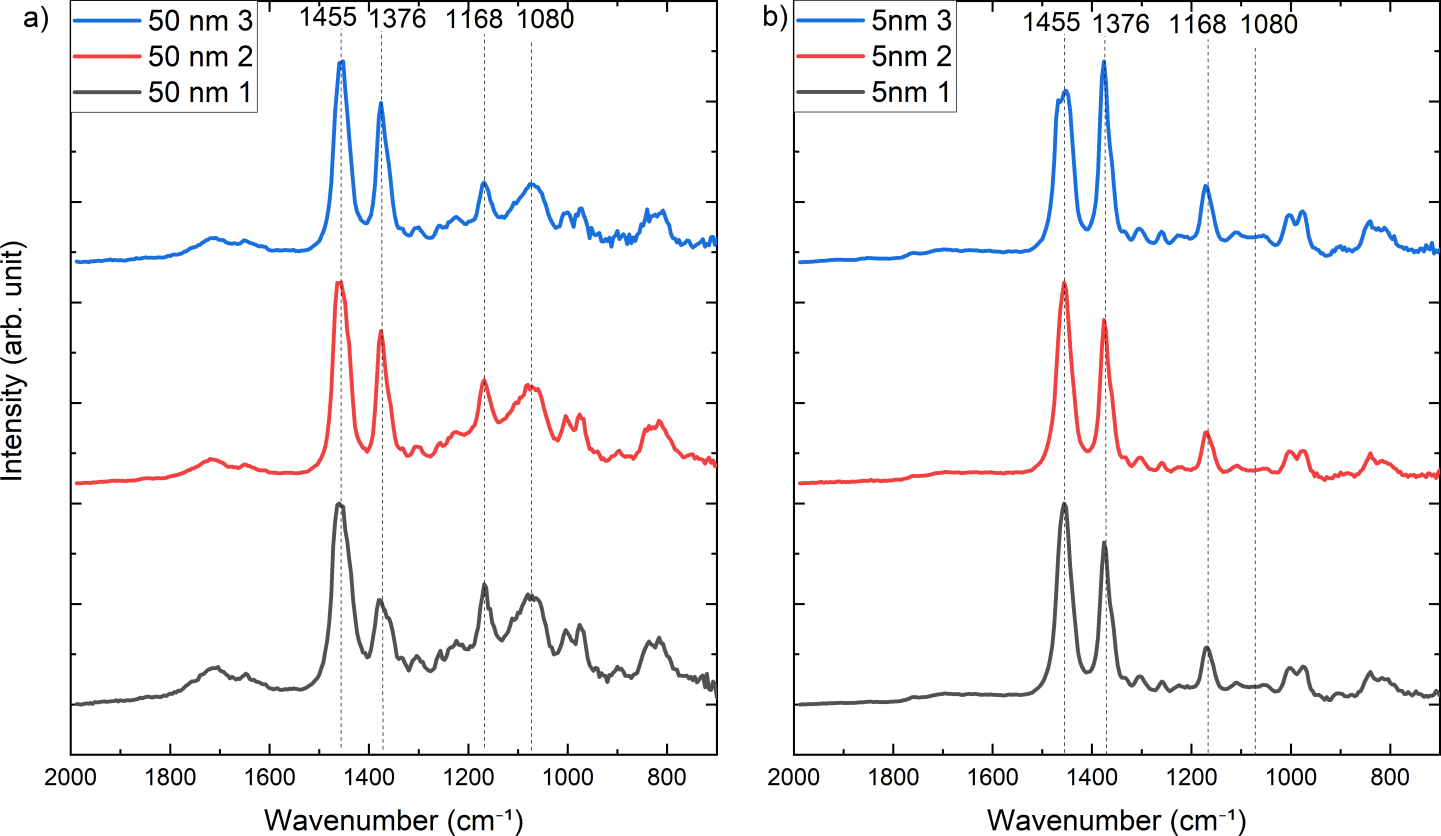
Contact mode AFM-IR spectra of each of the polypropylene samples with 50 nm (a) and 5 nm SiO*_x_* (b) recorded in the AFM image in Figure 4.

**Figure 6:**
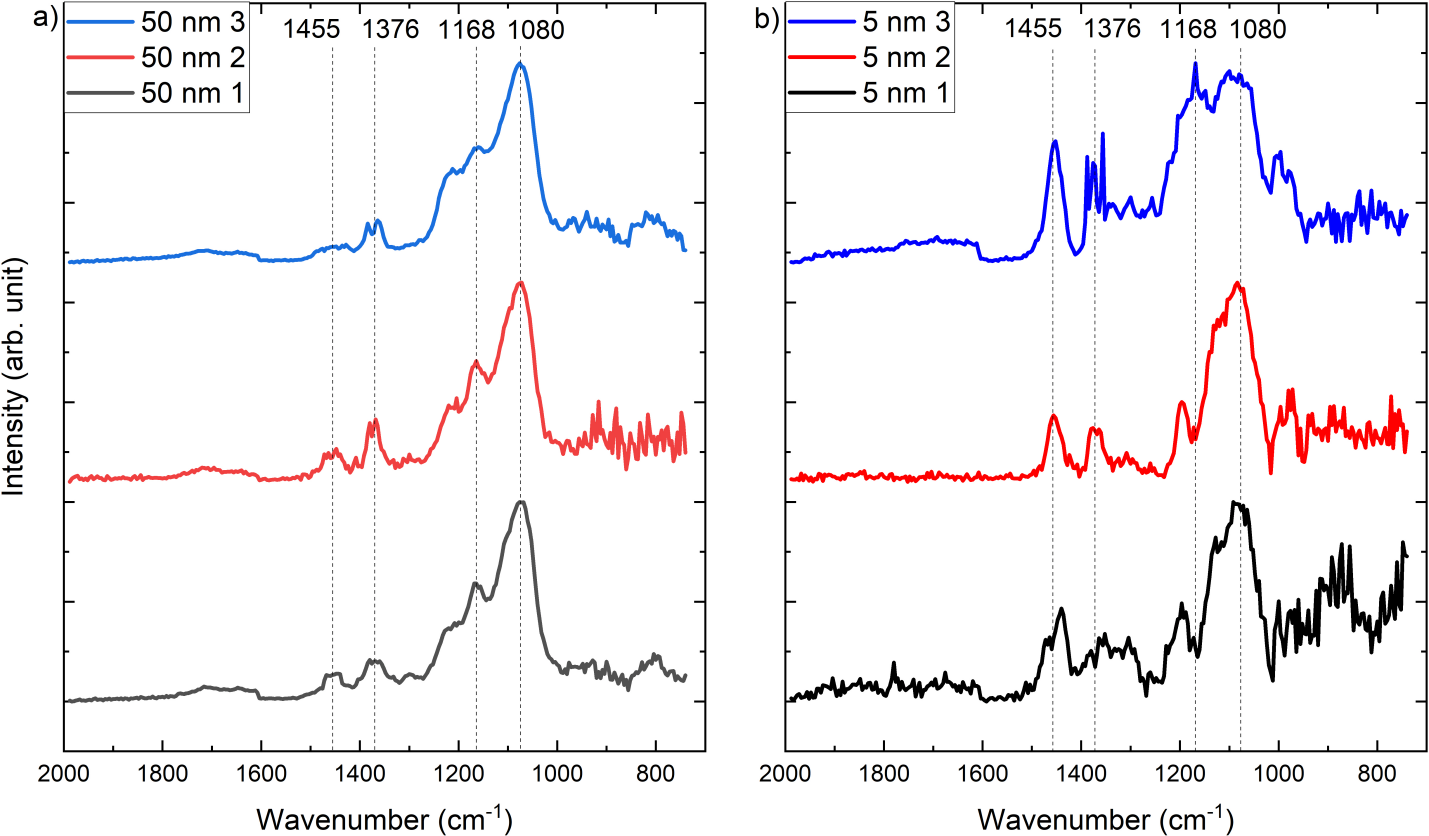
Surface-sensitive mode AFM-IR spectra of each of the polypropylene samples with 50 nm (a) and 5 nm thin PECVD SiO*_x_* film (b) recorded in the AFM image in Figure 4.

The photothermal AFM-IR spectra in contact and surface-sensitive mode were collected at the spots marked in the AFM images, shown in Figure 4. The resulting spectra are shown in Figure 5 (contact mode) and Figure 6 (surface-sensitive mode). The spectra are normalized for a better comparison.

The contact mode AFM-IR spectra in Figure 5 show peaks according to CH_3_ asymmetric deformation vibration and CH_2_ bending at 1455 cm^−1^, CH_3_ symmetric deformation vibration at 1376 cm^−1^, and CH_3_ rocking bands at 1168 cm^−1^, all of which originate from the polymer foil [21–22]. In the spectra of the sample with a 50 nm SiO*_x_* film, a broad peak occurs around 1080 cm^−1^, which corresponds to Si–O–Si transversal oscillation modes [23–25]. In contrast, the sample with the thin SiO*_x_* film shows no broad peak around 1080 cm^−1^. Nevertheless, the XPS spectra in Figure 3 have shown that the SiO*_x_* deposition was successful. Therefore, these results indicate that the sensitivity limit of photothermal AFM-IR spectroscopy in contact mode is reached due to the small thickness of the deposited SiO*_x_* film.

To improve the chemical sensitivity of the 5 nm SiO*_x_* film, the surface-sensitive mode was employed (see Figure 6). Here, photothermal AFM-IR spectra were collected on the same samples; however, this time a drive frequency of 847 kHz and a detection frequency of 205 kHz were used. In addition, the laser was tuned to 646 kHz.

The surface-sensitive mode AFM-IR spectra in Figure 6 show a high intensity in the area between 1000 and 1200 cm^−1^, while the intensity of the CH_3_ and CH_2_ absorption bands at 1455 cm^−1^ and 1376 cm^−1^ weakened. In the surface-sensitive mode, the measured signal contains less information of the deeper bulk phase of the substrate and more information of the near-surface region. In samples with a thicker layer of SiO*_x_*, this results in an improved signal-to-noise ratio. Even though the spectra of the sample with a low SiO*_x_* thickness still show sizable peaks originating from the substrate underneath the SiO*_x_* layer, improved surface sensitivity is achieved for both samples. Upon a closer look at the spectra, the broad peak corresponding to the TO Si–O–Si vibration at 1080 cm^−1^ can be clearly identified and even dominate the overall spectra [23,26].

The peak at 1168 cm^−1^ corresponding to the CH_3_ rocking is superposed to the broad Si–O–Si band, as can be seen in all spectra measured for the sample with the 50 nm SiO*_x_* layer. Interestingly, this peak appears as a negative peak in some of the spectra measured for the 5 nm SiO*_x_* sample (spectra 1 and 2) of the sample with a 5 nm SiO*_x_* layer (Figure 6b, black and red spectra). It is unclear at present whether this effect is due to a tip-induced artifact.

It is interesting to note that the relative intensity of the polypropylene band at 1455 cm^−1^ decreases when the 50 nm SiO*_x_* sample is measured in the surface-sensitive mode. The relative intensity of this band increases again in the measurement of the 5 nm SiO*_x_* layer. The results seem to indicate that the band at 1455 cm^−1^ could be associated to bulk regions within the polypropylene, further away from the interface to the SiO*_x_*.

To highlight the advantage of the surface-sensitive mode and to show the homogeneity of the coating, further measurements on the sample with the 5 nm SiO*_x_* coating were performed. Therefore, a comparison of surface-sensitive AFM-IR using contact mode AFM-IR and with the laser tuned to a higher contact resonance mode for a higher surface sensitivity was done.

**Figure 7:**
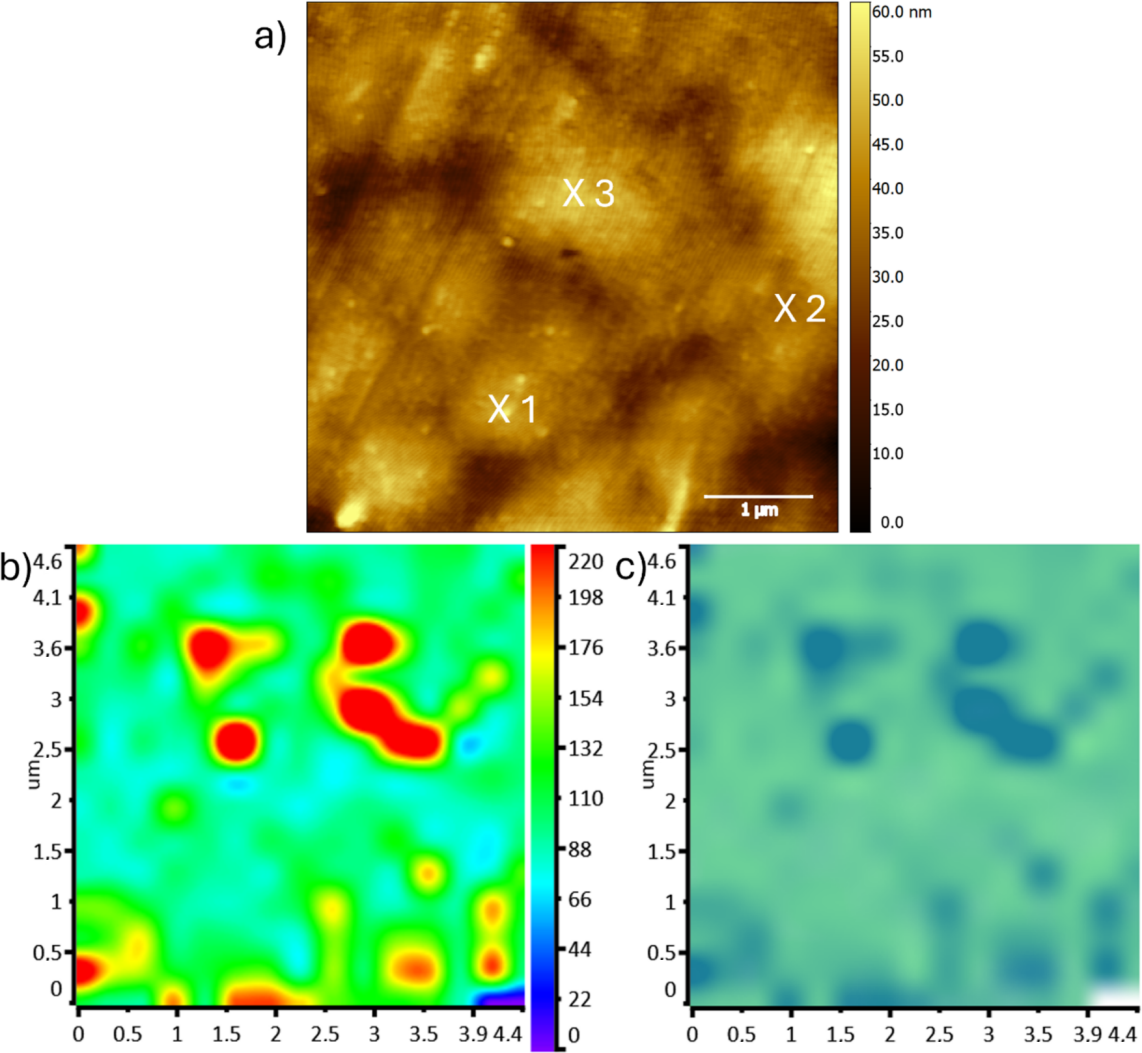
a) Height image recorded in contact mode of the investigated area of the 5 nm thin SiO*_x_* film coated sample. The markings in this image correspond to the spots where the AFM-IR spectra were collected. Images b) and c) show the hyperspectral image of the area measured with 15 × 15 single IR-spectra recorded with a laser pulse tuned to 771 kHz in contact mode. Image b) shows the intensity distribution of the 1080 cm^−1^ absorption band. In image c) the intensity ratio of the absorption bands at 1080 cm^−1^ (blue) and 1455 cm^−1^ (green) are shown.

The topography image in Figure 7a shows a surface with elevations and valleys. The hyperspectral measurement of this area indicates an overall signal of the Si–O–Si stretching band at 1080 cm^−1^ with some islands with a higher intensity. These can originate from small differences in the film thickness. The hyperspectral image (Figure 7c) shows the ratio of the Si–O–Si stretching band at 1080 cm^−1^ (blue) and the absorption band according to the CH_3_ asymmetric deformation vibration and the CH_2_ bending at 1455 cm^−1^ (green). As expected, the intensity of the polypropylene peak over the full image is more intense. It must be noted that, at the end of the measurement, the last two spectra showed no signal which can be seen in the hyperspectral images in the lower right corner.

In addition to the hyperspectral image, single AFM-IR spectra were recorded at selected spots shown in Figure 7a. Here, the spectra were collected in surface-sensitive mode with a drive frequency of 764 kHz and a detection frequency of 195 kHz. The laser pulse was tuned to 566 kHz, as well as in contact mode with a laser pulsed corresponding to the higher contact resonance mode at 771 kHz. The images reveal that at such small thickness the film is not perfectly homogeneous.

**Figure 8:**
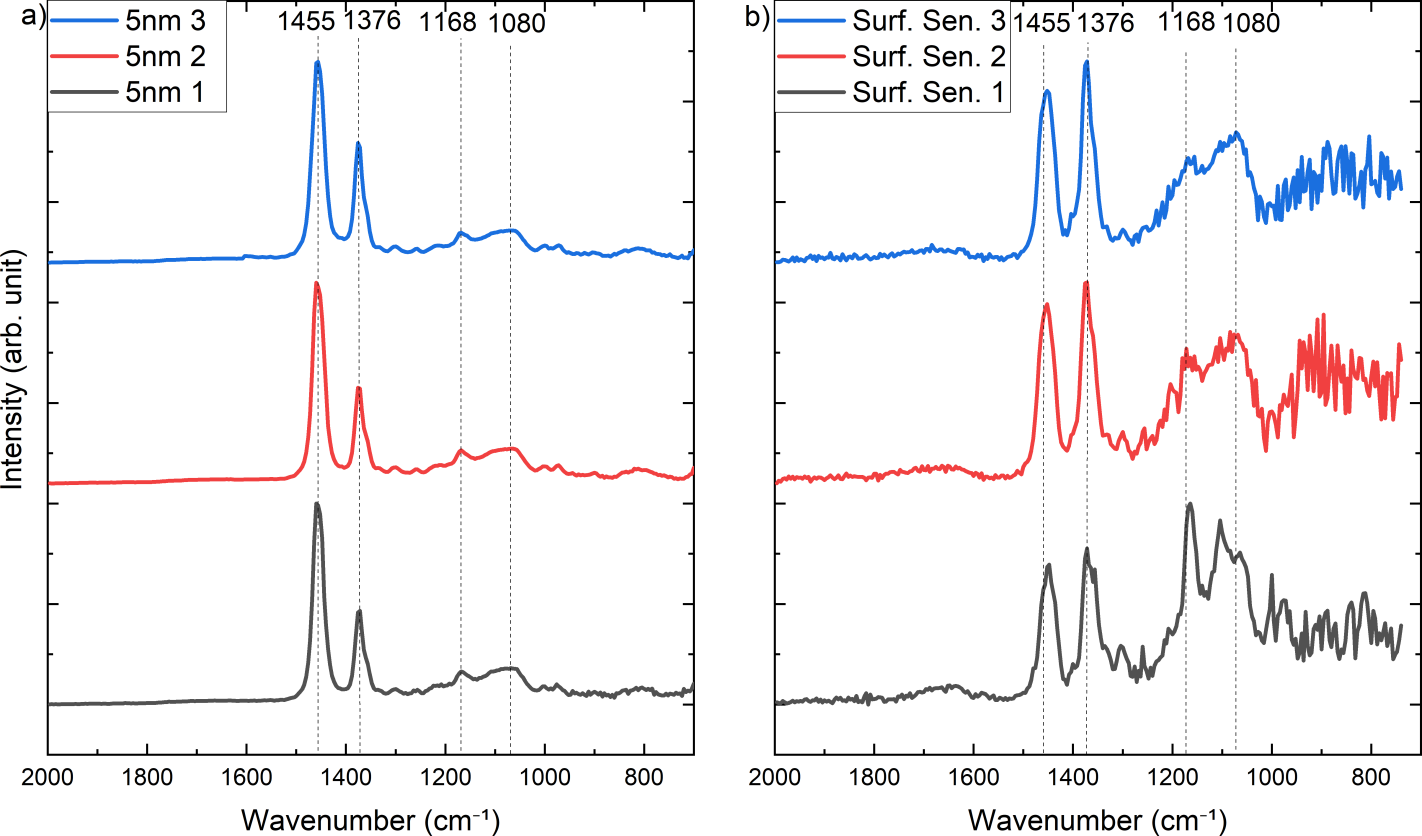
AFM-IR spectra collected in contact mode with a laser pulse frequency of 771 kHz corresponding to the fourth eigenmode frequency of the cantilever (a). In b) the AFM-IR spectra collected in surface-sensitive mode with a drive frequency of 764 kHz, a detection frequency of 195 kHz, and a laser pulse frequency of 566 kHz are shown.

The AFM-IR spectra in Figure 8a show the measurement in contact mode. The peaks corresponding to the polypropylene substrate at 1455 cm^−1^, 1376 cm^−1^, and 1168 cm^−1^ are clearly visible. Additionally, in these measurements with the laser pulse tuned to 771 kHz, the peak of the Si–O–Si layer at 1080 cm^−1^ is also detectable. This is a significant improvement compared to the previous spectra recorded in contact mode where the laser pulse was set to 69 kHz. Nevertheless, the spectra in Figure 8b were recorded in surface-sensitive mode and show the potential of this technique. The signal of the 5 nm thin SiO*_x_* layer has increased enormously. These measurements show the high sensitivity of this technique by detecting even 5 nm ultra-thin coatings on top of a polymeric substrate. This effect could have been supported by the choice of substrate material as shown in the work of Rosenberger et al. [27], where a polymeric substrate supports the sensitivity of AFM-IR detecting thin carbon nanotubes. Nevertheless, the system shown here is not completely transferable, as samples with a continuous coating were examined in this study.

These results highlight the potential of the AFM-IR surface-sensitive mode for the investigation of ultra-thin films and the interfaces to the supporting materials.

## Conclusion

The benefits of the surface-sensitive mode in the AFM-IR characterization of thin PECVD SiO*_x_* films on polymer substrates were investigated in this study. Our results exemplify the enormous improvements in thin film sensitivity that can be achieved by this mode in comparison to the established contact mode measurements. While the measurements in contact mode were dominated by the signal of the polypropylene substrate, we were able to record clear and well-defined signals of the SiO*_x_* thin films in surface-sensitive mode. This way, we were able to record AFM-IR spectra of a 5 nm thin PECVD SiO*_x_* layer grown on the polypropylene substrate.

AFM-IR enabled for the first time the analysis of PECVD SiO*_x_* thin film inhomogeneities during the initial layer growth.

Overall, this work demonstrates the significant improvement in sensitivity of the surface-sensitive mode for AFM-IR near-surface measurements of thin films.

## Data Availability

The data that supports the findings of this study is available from the corresponding author upon reasonable request.
