# Correction: AFM-IR investigation of thin PECVD SiO*_x_* films on a polypropylene substrate in the surface-sensitive mode

**DOI:** 10.3762/bjnano.16.19

**Published:** 2025-02-20

**Authors:** Hendrik Müller, Hartmut Stadler, Teresa de los Arcos, Adrian Keller, Guido Grundmeier

**Affiliations:** 1 Technical and Macromolecular Chemistry, Paderborn University, Warburger Str. 100, 33098 Paderborn, Germany; 2 Bruker Nano Surfaces and Metrology Division, Östliche Rheinbrückenstr. 49, 76187 Karlsruhe, Germany

**Keywords:** AFM-IR, polypropylene, surface-sensitive mode, silicon oxide, thin films, XPS

The authors regret that the acknowledgement in the publication is unfortunately not complete.

The following sentence in the Funding section is missing: This work was supported by the German Research Foundation (DFG) under grant number 407752136 and North Rhine-Westphalia based on the funding for large appliances (AFM-IR).

The complete funding section should read:

